# Induction of long-term B-cell depletion in refractory rheumatoid arthritis patients preferentially affects autoreactive more than protective humoral immunity

**DOI:** 10.1186/ar3770

**Published:** 2012-03-12

**Authors:** YK Onno Teng, Gillian Wheater, Vanessa E Hogan, Philip Stocks, EW Nivine Levarht, Tom WJ Huizinga, Rene EM Toes, Jacob M van Laar

**Affiliations:** 1 Department of Rheumatology, C1-R, Leiden University Medical Center, PO Box 9600, NL-2300 RC Leiden, The Netherlands; 2 Department of Biochemistry, The James Cook University Hospital, Marton Road, Middlesbrough, Cleveland, TS4 3BW, UK; 3 Musculoskeletal Research Group, Institute of Cellular Medicine, Newcastle University, 4th Floor Cookson Building, The Medical School, Framlington Place, Newcastle upon Tyne, NE2 4HH, UK

## Abstract

**Introduction:**

B-cell depletion has become a common treatment strategy in anti-TNF-refractory rheumatoid arthritis (RA). Although the exact mechanism of how B-cell depletion leads to clinical amelioration in RA remains to be elucidated, repetitive treatment with B-cell-depleting agents leading to long-term B-cell depletion has been reported to be beneficial. The latter has led to the hypothesis that the beneficial effects of B-cell depletion might act through their influence on pathogenic autoreactive plasma cells.

**Methods:**

In this study, we investigated the effects of a fixed retreatment regimen with anti-CD20 mAbs on the humoral (auto)immune system in a cohort of therapy-refractory RA patients.

**Results:**

Fixed retreatment led to long-term B-cell depletion in peripheral blood, bone marrow and, to a lesser extent, synovium. Also, pathologic autoantibody secretion (that is, anticitrullinated peptide antibodies (ACPAs)) was more profoundly affected by long-term depletion than by physiological protective antibody secretion (that is, against measles, mumps and rubella). This was further illustrated by a significantly shorter estimated life span of ACPA-IgG secretion compared to total IgG secretion as well as protective antibody secretion.

**Conclusion:**

By studying plasma cell function during an extensive 2-year period of B-cell depletion, autoantibody secretion was significantly shorter-lived than physiologically protective antibody secretion. This suggests that the longevity of autoreactive plasma cells is different from protective long-lived plasma cells and might indicate a therapeutic window for therapies that target plasma cells.

## Introduction

Rheumatoid arthritis (RA) is a chronic autoimmune disease typically characterised by symmetrical polyarthritis, joint destruction and an impaired quality of life. RA patients are generally treated with disease-modifying antirheumatic drugs (DMARDs) and, when treatment fails, with biologicals, including inhibitors of TNFα (anti-TNFα) [1]. Recommendations for the use of other biologicals, notably for B-cell-depleting mAbs, propagate their use to RA patients in whom treatment with TNF-depleting monoclonal antibodies has failed [2]. Several clinical studies have demonstrated the efficacy of B-cell depletion in RA [3-5]. As the therapeutic effects of a single treatment course are transient in the majority of patients, repeat treatment with B-cell-depleting agents is necessary to maintain efficacy [6-8]. The rationale for depleting B cells in RA is based on the role of the humoral immune system in the pathogenesis of RA. The latter is supported by clinical studies showing that seropositive (for rheumatoid factor (RF) and anticitrullinated protein antibodies (ACPAs)) RA patients, who have more severe disease and a worse prognosis [9,10], respond better to B-cell-depleting therapy. Thus far whether the beneficial effects of B-cell depletion act through their influence on pathogenic autoreactive plasma cells remains speculative [11-13]. It has been shown that after one course of rituximab, serum concentrations of ACPA-immunoglobulin G (IgG) and RF-IgM decrease specifically, in contrast to antibodies against tetanus toxoid [3,14]. These observations can be explained by the direct cytotoxic effects of rituximab on CD20^+ ^short-lived plasmablasts, in contrast to CD20^- ^long-lived plasma cells. Importantly, although serum concentrations decreased, autoantibody secretion was not abrogated completely, which is indicative of persisting autoreactive plasma cells. As a consequence, targeting these long-lived autoreactive plasma cells has been a recent focus of clinical research [15]. Induction of long-lasting B-cell depletion is one possible way to target plasma cells indirectly by inhibiting B-cell differentiation and thus the production of long-lived plasma cells.

Therefore, in the present study, we investigated a population of RA patients in whom B-cell depletion was achieved for a period of at least 2 years (further referred to as 'long-term B-cell depletion') and studied its effects on the humoral (auto)immune system. This study was part of an open-label feasibility study in which refractory RA patients were treated with a regimen of fixed repeat treatment with rituximab as a means to achieve persistent B-cell depletion during the 2-year study period. Our aim in this proof-of-principle study was to investigate whether plasma cells, either autoreactive or protective, were directly or indirectly affected by long-term B-cell depletion. To this end we analysed blood, bone marrow and synovium to examine the extent of B-cell depletion and its effects on the secretion of RA-specific autoantibodies as well as physiological protective antibody secretion.

## Methods

### Study design

The present study involved paired samples of blood, bone marrow and synovium from 11 patients with severe RA who were positive for IgM rheumatoid factor (RF-IgM) and IgG autoantibodies against cyclic citrullinated peptides (ACPA-IgG). These patients were selected from among a cohort of 28 RA patients refractory to TNFα inhibitors who participated in a single-centre, open-label, phase I/II trial to investigate the safety, feasibility and efficacy of maintenance treatment with rituximab as described previously [16]. Seventeen patients were excluded from this study for the following reasons: three patients were excluded because of incomplete sampling, five patients because of the absence of RF-IgM and ACPA-IgG and nine patients dropped out of the study before 24 months (four patients because of infections, five patients for miscellaneous reasons: one serious infusion reaction, one accident requiring surgery, one inefficacy, one death and one lost to follow-up). The study protocol was approved by the Ethics Committee of the Leiden University Medical Center, and all patients gave their written informed consent.

### Study patients

The patients' median age was 54 years (range: 33 to 64), eight patients (73%) were female and the median disease duration was 12.4 years (range: 1.3 to 32.3). Patients had used a median of four DMARDs (range: two to six) and one TNF-blocking agent (range: zero to three). (One patient had a relative contraindication to TNF-blocking agents due to a history of B-cell lymphoma.) The patients' current medications included methotrexate (median dose of 15 mg/week (range: 2.5 to 25.0) in 11 patients) and oral prednisolone (median dose of 8.75 mg/day (range: 2.5 to 20.0) in 6 patients).

The median Disease Activity Score in 28 joints (DAS_28_), as assessed by the four-variable DAS_28 _according to the European League Against Rheumatism guidelines, was 6.15 (range: 3.01 to 7.67). The median erythrocyte sedimentation rate (ESR) was 49 mm/first hour (range: 5 to 134), and the median C-reactive protein (CRP) level was 24 mg/L (range: 2 to 110). The median Health Assessment Questionnaire (HAQ) score was 1.38 (range: 0.12 to 2.25), as assessed using the HAQ to quantify patients' functional disability. The median Sharp/van der Heijde score, which is used to assess joint damage based on X-rays of the hands and feet, was 51 (range: 20 to 164).

### Synovial tissue analysis

Arthroscopy of clinically affected knees and sampling of synovial tissue specimens was performed in all patients. Synovial tissue specimens were stored in formalin and embedded in paraffin. Paraffin-embedded sections were cut for staining, encoded and semiquantitatively scored as previously described [16].

### Flow cytometric analysis

Blood samples and bone marrow aspirates (anticoagulated by using ethylenediaminetetraacetic acid) were obtained as described previously [16]. According to a stain-wash-no-lyse protocol, an estimated 500 × 10^3 ^cells were incubated with mouse anti-human mAbs, as specified below, in PBS/1% BSA at room temperature for 15 minutes. Next, fluorescence-activated cell sorting (FACS) lysing solution (BD Biosciences, San Jose, CA, USA) was used to lyse erythrocytes, after which cells were analysed immediately with a FACSCalibur flow cytometer (BD Biosciences). To allow quantification of cell numbers in each sample, 10^4 ^Flow-Count Fluorospheres (Beckman Coulter, Miami, FL, USA) were added immediately before flow cytometric analysis. The frequencies within the lymphocyte population were calculated using FlowJo software (Tree Star, Inc, Ashland, OR, USA). The detection limits were 1 × 10^6 ^cells/L in peripheral blood and 1% of lymphocytes in bone marrow. The following mAbs were used: anti-CD19-fluorescein isothiocyanate (H1B19), anti-CD19-phycoerythrin (anti-CD-19-PE; H1B19), anti-CD19-PerCP-Cy5.5 (SJ25C1) and anti-CD3-APC (UCHT1) (all from BD Biosciences).

### Flow cytometric cell sorting

Bone marrow mononuclear cells (BMMCs; 30 × 10^6^) were stained for flow cytometric cell sorting with mouse anti-human mAbs in PBS/1% BSA for 30 minutes at 4°C in the dark. The following mAbs were used: anti-IgD-PE (IAG-2), anti-CD38-PerCP-Cy5.5 (HIT-2) and anti-CD3-APC (UCHT1). Thereafter BMMCs were thoroughly washed and resuspended in PBS/1% BSA and immediately sorted into Iscove's modified Dulbecco's medium (IMDM)/50% FCS by using a FACSAria flow cytometer (BD Biosciences). Sorted cell populations were then titrated into ELISPOT plates (BD Biosciences) as mentioned below.

### Enzyme-linked immunosorbent spot assays

Ninety-six-well microtitre plates were coated with 100 μl/well goat anti-human poly-Ig (15 μg/ml) and PBS as control. The plates were incubated overnight at 4°C. They were then washed twice with PBS and blocked with 200 μl/well culture medium (IMDM + 10% FCS + 200 mM L-glutamine + 100 μg/ml penicillin/streptavidin) for 2 hours at 37°C in a 5% CO_2 _atmosphere. FACS cell populations were titrated into the ELISPOT plates in duplicate wells, and the plates were then incubated at 37°C in a 5% CO_2 _atmosphere overnight. The following day cells were discarded and washed from the plates with PBS/0.05% Tween 20 and tap water. Spots were visualised by detection with alkaline phosphatase-conjugated goat anti-human IgG, IgM or IgA, followed by substrate 5-bromo-4-chloro-3-indolyl phosphate/nitro blue tetrazolium (Sigma-Aldrich, St Louis, MO, USA) at 100 μl/well. Enzyme-linked immunosorbent spots (Elispots) were analysed using a stereomicroscope (Bioreader 3000 Pro; BIO-SYS GmbH, Karben, Germany), and the number of spots detected was plotted against the titrated number of cells per well, resulting in a sigmoid-shaped curve. The representative number of spots was selected from the linear part of the curve, then the data were presented as the mean frequency of IgG-, IgM- and IgA-secreting cells/10^6 ^mononuclear cells.

### Measurements of serum antibody concentrations

Serial serum samples taken from each patient were analysed for concentrations of total immunoglobulins, autoantibodies and protective antibodies. Total serum concentrations of IgG and IgM were measured by immunoturbidimetry on the COBAS INTEGRA 400 700 800 (Roche Diagnostics, Indianapolis, IN, USA) according to the manufacturer's guidelines.

Serum concentrations of RF-IgM were measured using a standardised ELISA as previously described [17]. Serum concentrations of ACPA-IgG, ACPA-IgM and ACPA-IgA were measured using a commercially available ELISA (Immunoscan RA Mark 2; Euro-Diagnostica, Arnhem, The Netherlands) according to the manufacturer's instructions and as previously described [18].

### Statistical analysis

Nonparametric Wilcoxon tests were used to compare follow-up concentrations of total immunoglobulins, autoantibodies and protective antibodies (measles, mumps and rubella) at baseline versus 6 months and at baseline versus 24 months. The lifetime of immunoglobulin-secreting cell populations was estimated by extrapolation of the measured serum concentrations during the 2 years of B-cell depletion. For each separate (auto)antibody, the consecutive median concentrations from 6 months to 24 months (thus excluding baseline values) were used to produce a linear fit curve, from which the lifetime was calculated (that is, the time point at which the fit curve crossed the *x*-axis). *P*-values less than 0.05 were considered significant.

## Results

### Fixed retreatment with rituximab induces long-term B-cell depletion in different tissue compartments of refractory rheumatoid arthritis patients

**Figure 1:**
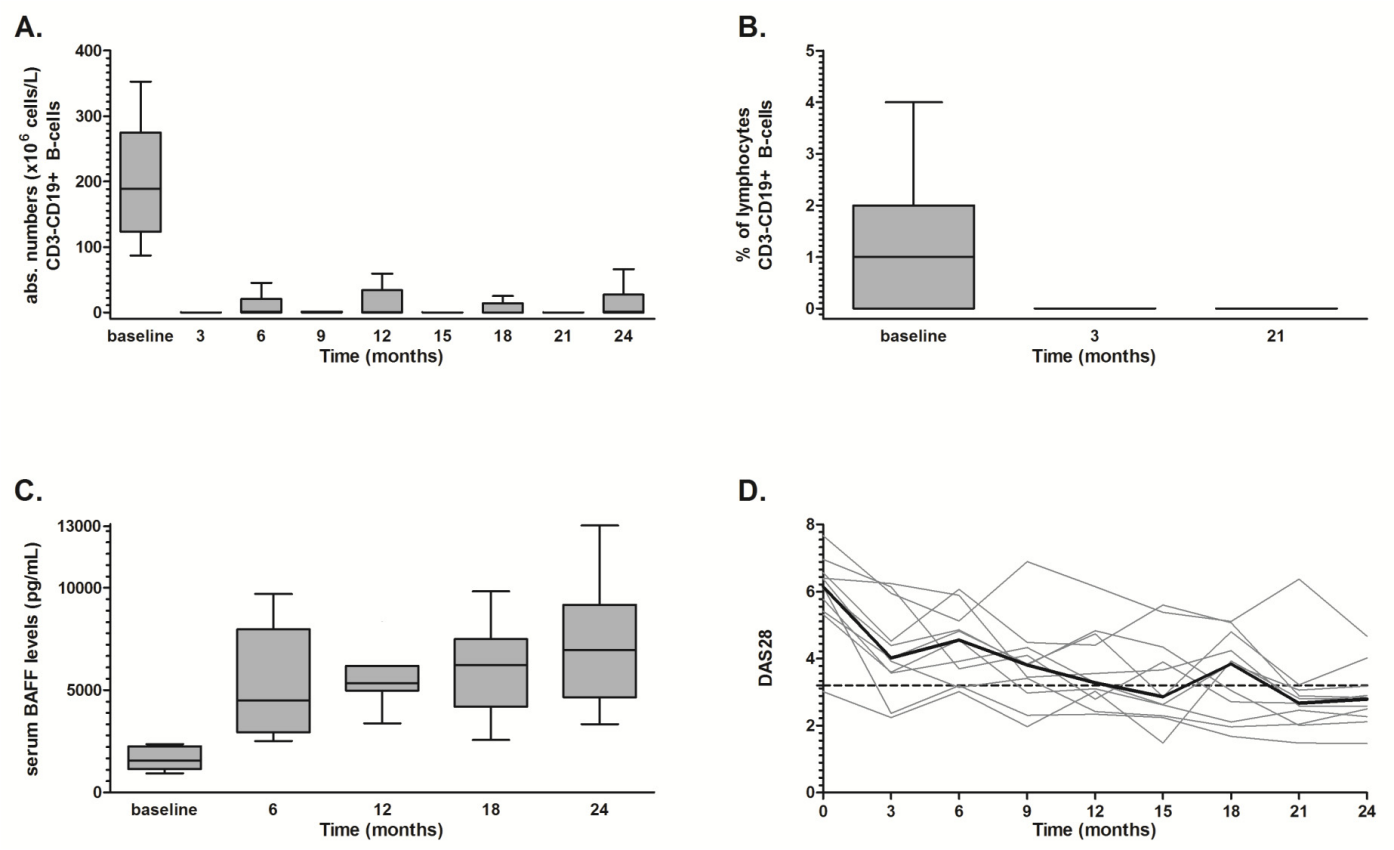
**Long-term B-cell depletion in peripheral blood and bone marrow in rheumatoid arthritis patients treated with a fixed regimen of 6-monthly anti-CD20 monoclonal antibodies with a follow-up of 24 months**. **(A) **Absolute numbers of CD3^-^CD19^+ ^B cells measured in peripheral blood every 3 months presented as Tukey box-and-whisker plots. **(B) **Percentages of CD3^-^CD19^+ ^B cells in bone marrow measured at baseline, 3 and 21 months presented as Tukey box-and-whisker plots. **(C) **Serum B lymphocyte-activating factor (BAFF) concentrations before every rituximab course presented as Tukey box-and-whisker plots. **(D) **Disease Activity Score in 28 joints (DAS_28_) of the 11 individual patients (grey lines) and overall median DAS_28 _scores (black line) measured every 3 months. A DAS_28 _score below 3.2 (dotted line) indicates low disease activity.

RA patients were treated with rituximab every 6 months in our prospective study, the clinical results of which have been published elsewhere [8]. The B-cell-depleting effects of this fixed retreatment protocol in peripheral blood and bone marrow are shown in Figure 1. We observed a significant reduction in absolute B-cell numbers in peripheral blood (Figure 1A). At baseline, patients had a median number of 189 × 10^6 ^cells/L (range: 87 to 353 × 10^6^) CD3^-^CD19^+ ^B cells. After each rituximab course, B-cell numbers fell to undetectable numbers as follows: In 10 of 11 patients at 3 months, B cells ranged from 0 to 1 × 10^6 ^cells/L; in 8 of 11 patients at 9 months, B cells ranged from 0 to 1 × 10^6 ^cells/L; in 10 of 11 patients at 15 months, B cells ranged from 0 to 1 × 10^6 ^cells/L; and in 10 of 11 patients at 21 months, B cells ranged from 0 to 4 × 10^6 ^cells/L. B-cell numbers started to rise about 6 months after each rituximab course, upon which patients were retreated with a subsequent course. Circulating numbers of B cells were persistently undetectable in 5 of 11 patients at 6 months (range: 0 to 425 × 10^6 ^cells/L), in 6 of 11 patients at 12 months (range: 0 to 59 × 10^6 ^cells/L), in 7 of 11 patients at 18 months (range: 0 to 113 × 10^6 ^cells/L) and in 5 of 11 patients at 24 months (range: 0 to 79 × 10^6 ^cells/L) (Figure 1A). Overall, five patients had persistently undetectable B-cell numbers during the complete 2-year follow-up. In bone marrow, CD3^-^CD19^+ ^B cells were effectively depleted from the baseline median 1% (range: 0% to 4%) of mononuclear cells to undetectable levels at 3 months and 21 months in all patients (Figure 1B).

Synovial tissue samples were collected from 9 of 11 patients at baseline and 3 months, which showed nonsignificant reductions of semiquantative scores for CD79a^+ ^B cells from a baseline median score of 2 (range: 0 to 4) to a median score of 2 (range: 0 to 3) (*P *= 0.41). Synovial tissue samples from six patients were available at 21 months, with the median score being 1 (range: 0 to 2; *P *= 0.70 compared to baseline). Importantly, CD20^+ ^cells were undetectable in all patients at 3 and 21 months. Nonsignificant changes were seen in CD138^+ ^plasma cells, with a median baseline score of 1 (range: 0 to 4) versus a median score of 1 at 3 months (range: 0 to 3; *P *= 0.49) and 0.5 at 21 months (*n *= 6 patients) (range: 0 to 2; *P *= 0.17 compared to baseline).

Significant increases in serum B lymphocyte-activating factor (BAFF) concentrations were observed after the first rituximab treatment. The baseline median concentration of 1,560 pg/ml (range: 940 to 2,361) increased to a median concentration of 4,499 pg/ml (range: 2,520 to 9,693) at 6 months (*P *= 0.005). Also, after 24 months, serum BAFF concentrations were significantly increased up to 6,950 pg/ml (range: 3,339 to 13,040) compared to 6 months (*P *= 0.005) (Figure 1C).

Clinically, patients had highly active disease, with the median DAS_28 _score being 6.15 (range: 3.01 to 7.67), which improved gradually to a median DAS_28 _score of 2.79 (range: 1.47 to 4.67) at the end of the 2-year study period (Figure 1D). A DAS_28 _score below 3.2 indicated low disease activity, which was achieved in 9 of 11 patients at the end of the study. One patient had a relapsing-remitting response to each rituximab treatment, and one patient showed improvement of severe disease (baseline DAS_28 _score 7.7) to moderate disease activity (DAS_28 _score 4.7). Median levels of CRP and ESR, used as indicators of inflammation, decreased from 24 mg/L (range: 2 to 110) to 4 mg/L (range: 2 to 31) and from 49 mm/first hour (range: 5 to 134) to 19 mm/first hour (range: 5 to 89), respectively.

### IgM, IgG and IgA secretion is derived from different CD3^-^CD38^bright ^cell populations in bone marrow

**Figure 2:**
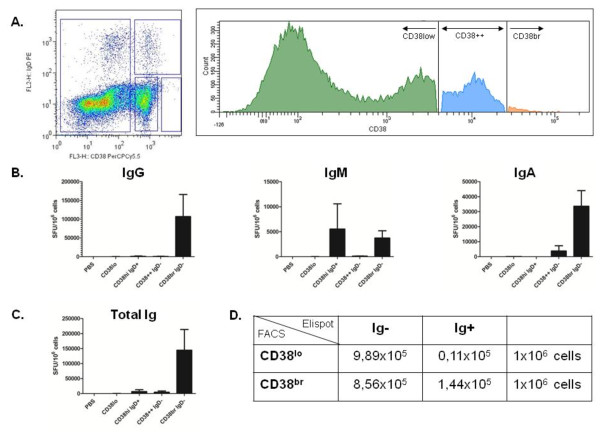
**Immunoglobulin secretion from CD3^-^CD38^bright ^cells in bone marrow**. **(A) **Fresh bone marrow-derived mononuclear cells (BMMCs) were stained with PerCP cyanin 5.5 dye for flow cytometric cell sorting (FL2H) according to immunoglobulin D (IgD) phycoerythrin and CD38 expression, resulting in four populations: CD38^low ^cells, IgD^+^CD38^high ^cells, IgD^-^CD38^2+ ^cells and IgD^-^CD38^bright ^cells. These cell populations were incubated on precoated ELISPOT plates as described in the Methods section. **(B) **Using the enzyme-linked immunosorbent spot assay, we detected the number of spot-forming units (SFUs) and quantified them for the IgG, IgM and IgA isotypes. PBS = phosphate-buffered saline. Bars represent means ± SEM of five experiments. **(C) **Calculated total number of SFUs for total immunoglobulin (total Ig) secretion. **(D) **2 × 2 table showing the sensitivity and specificity of the population of CD3^-^CD38^bright ^cells to secrete immunoglobulins. The sensitivity was 92.9%, and the specificity was 53.6%. FACS = fluorescence-activated cell sorting.

Next, we aimed to identify by flow cytometry the cell populations in bone marrow which were responsible for IgG, IgA and IgM secretion. Therefore, we investigated immunoglobulin secretion of cell populations sorted by flow cytometry on the basis of CD38 and IgD expression in conjunction with Elispot analysis (Figure 2A). The results are expressed as spot-forming units (SFU)/10^6 ^cells. In the CD3^-^CD38^bright^IgD^- ^cell population, we detected predominantly IgG-secreting cells (mean ± SEM: 107,067 ± 58,880 SFU/10^6 ^cells) and IgA-secreting cells (mean ± SEM: 33,667 ± 10,364 SFU/10^6 ^cells). A minority of CD3^-^CD38^bright^IgD^- ^cells were IgM-secreting cells (mean ± SEM: 3,733 ± 1,444 SFU/10^6 ^cells) (Figure 2B). In the CD3^-^CD38^bright^IgD^+ ^cell population, only IgM-secreting cells were detected (Figure 2B). With respect to the overall population of immunoglobulin-secreting cells, we observed significantly less SFUs in the cell populations with lower CD38 expression. The majority of immunoglobulin-secreting cells were found in the CD38^bright ^cell populations, with a minor fraction of IgA-secreting cells in the CD38^2+ ^cell population (Figure 2C). To further illustrate this finding, we calculated that the sensitivity for detecting immunoglobulin secretion in CD3^-^CD38^bright^IgD^- ^cells was 92.9% (Figure 2D).

Taken together, these data confirm that, in bone marrow, IgG and IgA secretion, as compared to IgM secretion, derive from different cell populations distinguishable by CD38 and IgD expression, presumably plasmablasts (CD3^-^CD38^bright^IgD^+^) and plasma cells (CD3^-^CD38^bright^IgD^-^), based upon the immunoglobulin isotypes secreted.

### Immunoglobulin G autoantibody secretion is preferentially affected during long-term B-cell depletion in comparison to protective immunoglobulin G antibody secretion

To evaluate the effects of long-term B-cell depletion on plasma cell-derived immunoglobulin secretion, serum concentrations of protective antibodies of the IgG isotype (that is, mumps, measles and rubella) were compared to serum concentrations of ACPA-IgG and to total IgG. As a control for plasmablast-derived immunoglobulin secretion, serum concentrations of total IgM, ACPA-IgM and RF-IgM, respectively, were measured. As shown in Table 1, serum concentrations of total IgG and IgM, as well as those of ACPA-IgG, ACPA-IgM and RF-IgM, decreased significantly after 6 months (effect of first treatment course) and also after 24 months (effect of the fixed retreatment regimen). Interestingly, total IgA and ACPA-IgA showed similar significantly decreasing trends after long-term B-cell depletion. These reductions were in contrast to the constant course of serum concentrations of measles-IgG, mumps-IgG and rubella-IgG over the entire 2 years of follow-up (Table 1).

**Table 1:** Effects of long-term B-cell depletion on serum concentrations of total immunoglobulin secretion, autoantibodies and protective antibodies^a^

	Baseline	6 months	12 months	18 months	24 months
Total IgG (g/L)	13.4 (5.4 to 16.5)	10.5 (5.4 to 15.0)^b^	10.4 (5.2 to 13.6)	9.35 (5.2 to 13.6)	9.15 (5.1 to 12.2)^c^
ACPA-IgG (U/ml)	423 (65.6 to 1116)	349 (47.0 to 647)^b^	243 (43.9 to 410)	215 (57.2 to 361)	199 (43.6 to 354)^c^
Measles-IgG (U/ml)	3.72 (2.43 to 4.71)	3.81 (2.75 to 5.03)	3.84 (2.53 to 5.03)	3.58 (2.63 to 5.04)	3.67 (2.81 to 4.94)
Mumps-IgG (U/ml)	3.16 (0.65 to 4.67)	3.31 (0.53 to 4.77)	3.31 (0.46 to 4.89)	3.33 (0.64 to 4.78)	3.62 (0.67 to 5.11)
Rubella-IgG (U/ml)	101 (50.2 to 303)	100 (29.7 to 310)	91.6 (20.5 to 333)	103 (25.6 to 311)	106 (26.9 to 301)
Total IgM (g/L)	1.7 (0.6 to 3.7)	1.2 (0.3 to 2.1)^b^	1.1 (0.2 to 2.2)	1.0 (0.2 to 1.9)	0.9 (0.2 to 2.0)^c^
ACPA-IgM (U/ml)	35.3 (23.4 to 101)	24.4 (16.3 to 72.9)^b^	21.4 (16.2 to 49.1)	19.8 (14.4 to 37.0)	20.0 (11.8 to 30.8)^c^
RF-IgM (U/ml)	76.0 (10.0 to 792)	32 (8.0 to 385)^b^	16 (5 to 555)	13 (2.0 to 310)	10 (1 to 208)^c^
Total IgA (g/L)	2.0 (1.2 to 5.5)	1.7 (1.0 to 5.7)^b^	1.8 (0.8 to 5.5)	1.6 (0.8 to 4.3)	1.55 (0.8 to 4.2)^c^
ACPA-IgA (U/ml)	20.7 (3.16 to 90.8)	12.5 (5.22 to 87.4)	9.79 (2.81 to 97.9)	8.49 (3.69 to 60.3)	8.50 (2.63 to 44.4)^c^

^a^ACPA = anticitrullinated peptide antibody; Ig = immunoglobulin; RF = rheumatoid factor. Data are medians (ranges). ^b^*P *< 0.05 (baseline versus 6 months). ^c^*P *< 0.05 (6 months versus 24 months).

**Figure 3:**
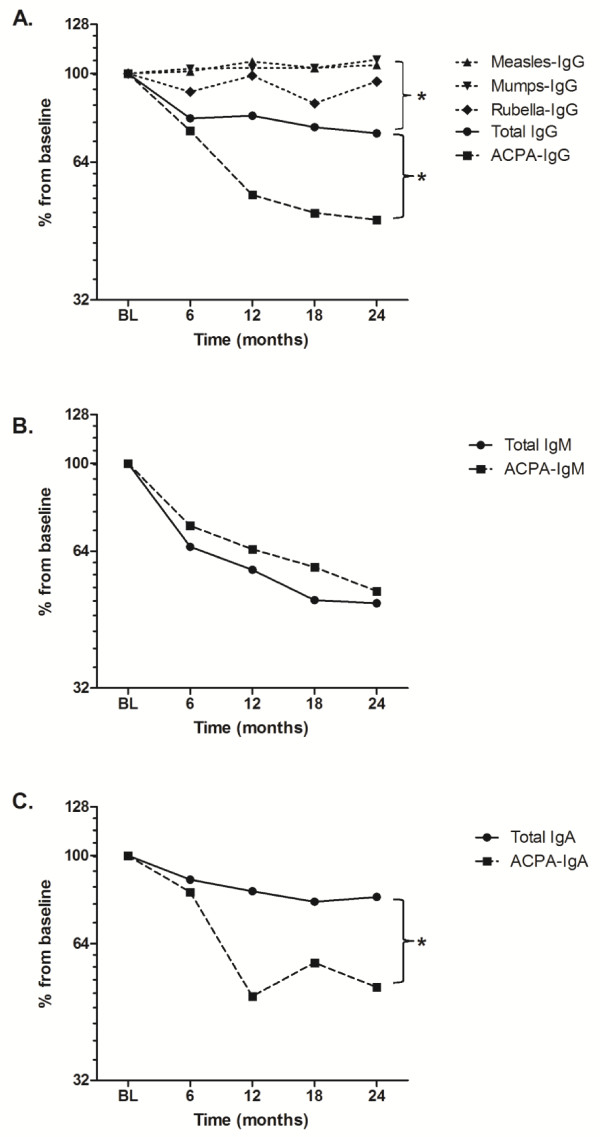
**Comparison of relative effects of long-term B-cell depletion between total immunoglobulin secretion, autoantibody secretion and protective antibody secretion**. Patients were treated with a fixed regimen of 6-monthly anti-CD20 monoclonal antibodies at baseline, 6, 12 and 18 months with a follow-up of 24 months. **(A) **Median percentage change from baseline serum concentrations of total immunoglobulin G (total IgG), rheumatoid arthritis (RA)-specific anticitrullinated protein autoantibodies of the IgG isotype (ACPA-IgG) and IgG-isotype antibodies against measles (measles-IgG), mumps (mumps-IgG) and rubella (rubella-IgG). **(B) **Median percentage change from baseline serum concentrations of total IgM and RA-specific ACPA-IgM. **(C) **Median percentage change from baseline serum concentrations of total IgA and RA-specific ACPA-IgA. Brackets in (A) through (C) represent the nonparametric statistical comparison at 24 months between the represented (auto)antibodies and total IgG/IgA. * *P *< 0.05. For clear representation, log_2 _scales are used for the *y*-axes.

To further unravel whether long-term B-cell depletion specifically reduced autoantibody secretion, the percentage change from baseline of total serum IgG was compared to the different IgG specificities (Figure 3). We found that the relative reduction from baseline of total IgG (median 74%, range: 52% to 107%) was significantly larger than the reductions of measles-IgG (median 104%, range: 77% to 120%; *P *= 0.004), mumps-IgG (median 107%, range: 78% to 121%; *P *= 0.003) and rubella-IgG (median 96%, range: 54% to 138%; *P *= 0.03). In addition, the relative reduction of ACPA-IgG (median 48%, range: 16% to 85%) was significantly larger than that of total IgG (median 74%, range: 52% to 107%) (*P *= 0.01) (Figure 3A). Illustratively, when assuming a B-cell-depleted state would be maintained, we estimated by means of extrapolation that the survival of ACPA-IgG-secreting cells was 9.1 years (linear fit curve, Spearman's ρ = 0.89) compared to an estimated lifetime of 38.3 years (Spearman's ρ = 0.89) for the total IgG-secreting cell population and infinite (∞) lifetime for measles-IgG, mumps-IgG and rubella-IgG (Table 2).

**Table 2:** Estimated life span of protective and autoreactive humoral immunity during long-term B-cell depletion^a^

		Individual data analysis	
		
	**Pooled data analysis**^**b**^	**Life span in years**^**c**^	
	
	**Median life span in years (Spearman's ρ)**^**d**^	Median (range)	**Spearman's ρ (range)**^**d**^
Total IgG	38.3 (0.89)	35.5 (10.1 to ∞)	0.75 (0.09 to 0.99)
ACPA-IgG	9.08 (0.89)	10.1 (5.22 to ∞)	0.83 (0.48 to 1.0)
Measles-IgG	∞ (0.66)	∞ (15.9 to ∞)	0.48 (0.01 to 0.92)
Mumps-IgG	∞ (0.90)	∞ (24.0 to ∞)	0.60 (0.04 to 0.84)
Rubella-IgG	75.5 (0.35)	153 (9.03 to ∞)	0.41 (0.04 to 0.93)
Total IgM	12.3 (0.96)	10.6 (6.0 to 36.7)	0.83 (0.38 to 1.0)
ACPA-IgM	11.7 (1.0)	14.9 (5.71 to ∞)	0.88 (0.07 to 0.99)
RF-IgM	4.78 (0.96)	5.27 (4.35 to 7.20)	0.94 (0.68 to 0.99)
Total IgA	33.5 (0.85)	22.8 (8.47 to ∞)	0.77 (0.26 to 0.95)
ACPA-IgA	9.48 (0.71)	9.33 (6.15 to 48.4)	0.88 (0.16 to 0.98)

^a^ACPA = anticitrullinated peptide antibody; Ig = immunoglobulin; RF = rheumatoid factor. ^b^Life span was estimated using the median data for all patients pooled together as depicted in Figure 3. ^c^Life span was estimated for each individual patient, here summarised in the depicted medians (ranges). ^d^Spearman's ρ data are correlation coefficients of the linear curves used to estimate the life span, which is explained in the Methods section.

As a control, the anticipated effects of long-term B-cell depletion on short-lived IgM-secreting plasmablasts were demonstrated by a persistent decrease in the percentage change from baseline total serum IgM and ACPA-IgM (Figure 4C). No significant differences were seen in the relative reductions between total IgM (median 52%, range: 31% to 84%) and ACPA-IgM (median 48%, range: 16% to 85%) (*P *= 0.27) (Figure 3B). This was also illustrated by the comparable estimated survival times of ACPA-IgM-secreting cells, 11.7 years (linear fit curve, *r*^2 ^= 0.99), and the total IgM-secreting cell population, 12.3 years (*r*^2 ^= 0.96) (Table 2). Of note, the relative reduction from baseline of RF-IgM at 24 months (median 13%, range: 5% to 30%; *P *= 0.003) was significantly more pronounced than other IgM-secreting plasmablasts, with an accordingly shorter estimated survival of RF-IgM-secreting cells of 4.8 years (*r*^2 ^= 0.96) (Table 2).

With respect to the IgA isotype, the relative reduction of ACPA-IgA (median 51%, range: 41% to 165%) was significantly larger than that of total IgA (median 81%, range: 44% to 92%) (*P *= 0.02) (Figure 3C). Again assuming persistence of B-cell depletion, we estimated that the survival times were 8.7 years (linear fit curve, *r*^2 ^= 0.94) for ACPA-IgA-secreting cells and 23.4 years (*r*^2 ^= 0.96) for the total IgA-secreting cell population (Table 2).

## Discussion

Our aim in this study was to investigate how long-term B-cell depletion affects the humoral immune system in refractory RA patients. We analysed data from a cohort of RA patients who received 6-monthly fixed repeat treatment with rituximab, which led to an extended period of 2 years of B-cell depletion in blood, bone marrow and, to a lesser extent, in synovium. We have shown that plasma cells secreting RA-specific autoantibodies were significantly more susceptible to this regimen than plasma cells secreting protective antibodies, thus indicating that the presence of differentiating CD20^+ ^B cells in the periphery is necessary to maintain pathogenic autoantibody secretion, in contrast to physiological protective antibody secreting.

In the present study, we investigated RA patients in whom an extended period of B-cell depletion during a 2-year period was achieved by applying a fixed retreatment protocol with rituximab, an anti-CD20 B-cell-depleting mAb [8]. Patients in whom prolonged periods of B-cell depletion in blood were observed after one treatment course of rituximab have previously been described; however, in most patients, one treatment course led to a transient period of 6 to 9 months of B-cell depletion measured in peripheral blood [7]. Repeated courses of rituximab have been reported previously; however, in all of those studies, a subsequent course of rituximab could be given only when B-cell numbers had returned in peripheral blood [7,19]. Herein we report on the depletory effects of a fixed repeat treatment regimen that led to persisting complete B-cell depletion in blood and bone marrow. Not surprisingly, B-cell depletion was temporary, as demonstrated by small increases in B-cell numbers in peripheral blood 6 months after every treatment, at which time patients received a retreatment course with rituximab. In synovium, depletion of CD79a^+ ^B cells was incomplete. This can be explained by the fact that CD79a is also expressed on morphological plasma cells [20,21]. Therefore, although CD20^+ ^B cells were eliminated in synovium, whether all CD79a^+ ^B cells were eliminated and only CD79a^+ ^plasma cells persisted remains unclear. Further confirmation that fixed retreatment led to long-term B-cell depletion was derived from serum BAFF concentrations, which significantly increased not only after the first treatment (as would be expected) but also after the fixed retreatment regimen. Fixed retreatment with anti-CD20 mAbs induced long-term B-cell depletion in our RA patients for at least the complete 2 years of study follow-up.

The treatment protocol of fixed retreatment was set up on the premise that eliminating autoreactive plasma cells through long-term B-cell depletion would ameliorate refractory disease in RA patients. This hypothesis was based on previous reports indicating that autoantibodies are produced by long-lived plasma cells as well as by short-lived plasmablasts [12,13]. The direct and indirect effects of long-term B-cell depletion on the Ig-secreting cell population (that is, plasmablasts and plasma cells) were studied through the serum concentrations of IgG, IgM and IgA (auto)antibodies. Flow cytometry was shown to be less reliable in determining the depleting effects of this regimen because of its low specificity (53.6%) for detecting bone marrow plasma cells and the lack of (auto)antigen specificity. Researchers in previous studies who have addressed the effect of B-cell-depleting therapy on autoantibody concentrations [14,16,22-26] have reported the effects of a single treatment with rituximab, to which long-lived plasma cells are known to be resistant. Moreover, repopulation of autoantibody-producing B-cell clones was not prevented [27]. In our present study, we have shown that the relative reduction in ACPA-IgG during long-term B-cell depletion was more pronounced than that of total IgG and, on the contrary, that serum concentrations of protective antibodies against measles, mumps and rubella remained constant. The latter was not unexpected, as these protective antiviral antibodies are produced by long-lived plasma cells that are present throughout life in humans [28]. Interestingly, within the first 6 months, we observed an initial decrease of total IgG as well as ACPA-IgG serum concentrations, reflecting direct elimination of a small, but relevant, fraction of CD20^+ ^IgG-producing cells, most likely early plasmablasts. The latter finding is supported by the concordant decrease in total IgM-, ACPA-IgM- and RF-IgM-producing plasmablasts. Of note, we observed a more rapid decline in RF-IgM secretion, which warrants further investigation.

Subsequent persisting B-cell depletion resulted in a significant reduction of ACPA-IgG as compared to total IgG, even though both are very likely secreted by CD20^- ^long-lived plasma cells, as evidenced by the complete depletion of all CD20^+ ^cells (be they B cells or plasmablasts) from bone marrow and synovium. Collectively, these data demonstrate that the effect of long-term B-cell depletion on humoral autoimmunity in RA is twofold: (1) direct elimination of autoreactive B cells, including ACPA-producing short-lived plasmablasts and (2) indirect interference in the differentiation of autoreactive B-cell towards ACPA-producing long-lived plasma cells.

Because of the observed indirect effects of continuous B-cell depletion on (auto)antibody secretion derived from mature, presumably long-lived plasma cells, we intended to further illustrate this observation by estimating the survival of ACPA-IgG secretion, total IgG secretion and protective antibody secretion (that is, measles-IgG, mumps-IgG and rubella-IgG). Extrapolation of the presented 2 years of follow-up data regarding antibody titres was done on the premise of two assumptions: (1) that B-cell depletion would be continued and the formation of new plasma cells was negligible and (2) that the decay of the plasma cell population followed a linear trend. Therefore, the estimated life span of (auto)antibody secretion reflected the senescence and extinction of established, long-lived plasma cells, which is in accord with the results of previous studies [28,29]. We estimated that the life span of ACPA-IgG secretion (9.08 years) was significantly shorter than the life span of the total IgG secretion (38.3 years) and those producing the protective antibodies against measles, mumps and rubella (75.5 years to ∞ years). The latter is in line with data previously reported by Amanna *et al*. [28], who demonstrated that B-cell triggering by viral antigens harbouring a highly repetitive antigenic structure and inducing cognate CD4^+ ^T-cell help led to differentiation into plasma cells with an increased life span, guaranteeing a lifelong antibody response [30]. Together these data convincingly illustrate that the autoantibody-secreting plasma cell population is 'less long-lived' and likely more dependent upon replenishment by newly formed B cells than the classically long-lived plasma cell population-producing protective antibodies. How to explain this different life span between pathogenic autoantibody secretion and physiological antibody secretion remains speculative: Either it can be explained by an intrinsic characteristic of autoreactive plasma cells that is traceable to the way autoreactive B cells were triggered [30] or it can be due to an inflammation-related reduction of extrinsic factors that support the survival of plasma cells [31,32], implying that the localisation of autoreactive plasma cells in synovium is crucial. Another explanation is that autoreactive plasma cells are more dependent upon continuous reactivation and differentiation of autoreactive B-cell [29] or that, in an even more complicated way, a combination of these models plays a role.

The clinical consequences of the observed susceptibility of autoantibody-producing plasma cells in the present study remain speculative. All patients showed improved disease activity during the study period, and we were unable to find any correlation between clinical response and the reduction of ACPA secretion. Recently, investigators in a large cohort study demonstrated the superiority of fixed retreatment (to a target DAS_28 _score below 2.6) with rituximab every 6 months compared to an on-demand strategy [33], confirming our hypothesis of a direct as well as indirect effect of prolonged B-cell depletion in RA. Therefore, when assuming that ACPA secretion is essential to the pathogenesis of RA, targeting autoreactive plasma cells by means of prolonged B-cell depletion makes sense biologically.

## Conclusion

In our present study, we have demonstrated that the autoantibody production in refractory RA patients was more susceptible to the long-term B-cell-depleting effects of fixed retreatment with anti-CD20 mAbs than protective antibody production. These results indicate that the homeostasis and sustainability of pathological autoantibody secretion is fundamentally different from physiological protective antibody secretion. Whether these data are indicative of the existence of a therapeutic window within which the autoreactive humoral immune response can be targeted without compromising the physiological long-lived humoral immune system requires further investigation.

## Abbreviations

ACPA: anticitrullinated peptide antibody; AID: autoimmune disease; BM: bone marrow; BMMC: bone marrow mononuclear cell; BSA: bovine serum albumin; CRP: C-reactive protein; DAS_28_: Disease Activity Score in 28 joints; DAS_44_: Disease Activity Score in 44 joints; DMARD: disease-modifying antirheumatic drug; ELISA: enzyme-linked immunosorbent assay; FCS: foetal calf serum; HAQ: Health Assessment Questionnaire; ISC: immunoglobulin-secreting cell; mAb: monoclonal antibody; MC: mononuclear cell; PB: peripheral blood; PBMC: peripheral blood mononuclear cell; PBS: phosphate-buffered saline; RA: rheumatoid arthritis; RF: rheumatoid factor; SLE: systemic lupus erythematosus; TNF: tumour necrosis factor.

## Competing interests

The work of YKOT was supported by an Agiko grant from the Netherlands Organization for Scientific Research. This study has been supported by the European Union-funded FP7-integrated project Masterswitch 223404. JMvL has received consultancy and speaker's fees and a research grant from Roche. The authors declare that they have no competing interests.

## Authors' contributions

YKOT contributed to the design of the study, acquisition of data, analysis and interpretation of data and manuscript preparation. VEH, GW, PS and EWNL contributed to the acquisition of data. REMT and TWJH contributed to the interpretation of data. JML contributed to the design of the study, analysis and interpretation of the data and preparation of the manuscript. All authors read and approved the final manuscript.
